# Highly Sensitive THz SPR Biosensor Based on Graphene-Coupled Prism Otto Structure

**DOI:** 10.3390/bios15090630

**Published:** 2025-09-21

**Authors:** Yu Xie, Zean Shen, Mingming Zhang, Mengjiao Ren, Wei Huang, Leyong Jiang

**Affiliations:** School of Physics and Electronics, Hunan Normal University, Changsha 410081, China; xieyu@hunnu.edu.cn (Y.X.); shenzean@hunnu.edu.cn (Z.S.); zhangmingming@hunnu.edu.cn (M.Z.); renmengjiao@hunnu.edu.cn (M.R.)

**Keywords:** optical biosensor, surface plasmon resonance, graphene

## Abstract

This study presents a theoretical investigation of a terahertz (THz) surface plasmon resonance (SPR) optical biosensor utilizing a graphene-integrated Otto configuration. Through systematic numerical simulations, we demonstrate that actively modulating graphene’s conductivity via an external magnetic field enables tunable SPR behavior with high phase sensitivity. The proposed sensor achieves a phase sensitivity of up to $3.1043\times 10^{5}$ deg RIU^−1^ in liquid sensing and $2.5854\times 10^{4}$ deg RIU^−1^ in gas sensing. This simulation-based work establishes a foundational framework for the development of highly sensitive, magneto-optically tunable optical sensors, highlighting their potential in chemical detection and medical diagnostics.

## 1. Introduction

An optical biosensor is a micro-nano scale device that transduces specific biomolecular interactions (e.g., antibody-antigen binding) into measurable optical signals, enabling highly sensitive and label-free detection of target analytes [1,2]. These sensors typically employ label-free detection, offering advantages such as non-contact operation, non-destructiveness, anti-interference, and minimal impact on target materials [3,4]. Consequently, they have been widely used in blood detection [5], heavy metal ion detection [6], pathogenic microorganism detection [7], and drug detection [8]. In recent years, advances in micro-nano processing and integration technologies have driven the evolution of biosensing toward micro-nano structures. Optical biosensors with micro-nano scale structure, such as microring cavities [9], photonic crystals [10], and optical waveguides [11], have garnered attention due to their small size and ease of integration. Among these, surface plasmon resonance (SPR) stands out as a label-free optical sensing technique with high sensitivity, real-time dynamic detection, low sample consumption and simple sample handling [12], finding applications in environmental monitoring [13,14], biomedicine [15,16], and food safety [17]. Notably, SPR is highly sensitive to environmental changes: even minor variations in the sensing medium can induce significant shifts in the resonance wavelength, angle, or phase [18], enabling the rapid development of high-sensitivity SPR-based biosensors in micro-nano sensing. In addition, femtosecond laser surface nanotexturing provides an alternative route for creating efficient terahertz (THz)-SPR structures [19]. Traditional SPR structures often use noble metals (e.g., Au [20], Ag [21]) to excite SPR. For example, Li et al. developed an SPR biosensor with gold nanoparticles as the sensing membrane, achieving a 50-fold sensitivity enhancement for ochre-toxin A detection [22]. Wang et al. designed a Ag/Au membrane SPR biosensor via silver mirror reaction on Au covered with a Ag membrane, which showed an 8-fold sensitivity increase compared to unmodified Au sensors in Hμman Immunoglobulin G (HIG) detection [23]. Zhang et al. fabricated a novel SPR biosensor using Fe_3_O_4_-Au nanocomposites, which significantly improved sensitivity for goat immunoglobulin detection [24]. However, metal-based SPR biosensors suffer from limited dynamic tunability [25], high loss [26], and operation restricted to the visible spectrum. Thus, exploring SPR biosensors in other wavelength bands to realize dynamic tunability and high sensitivity is both meaningful and necessary.

Over the past few years, two-dimensional (2D) nanomaterials, represented by transition metal disulfide (TMD) [27] such as MoS_2_ [28,29], WS_2_ [30], and graphene [31], have opened new avenues for optical biosensor research. Among them, graphene exhibits exceptional optical properties, including tunable conductivity, ultra-high carrier mobility, and low loss [32]. And its dielectric constant and conductivity can be dynamically modulated by adjusting carrier concentration via external voltage [33]. In the THz band, graphene conductivity can also be tuned by external magnetic fields, resulting in phenomena like giant Faraday rotation [34] and the quantum Hall effect [35], which are valuable for nanodevice development [36]. These properties grant graphene unique advantages in optical biosensors. For instance, Wu et al. proposed a graphene-based SPR biosensor with nearly 25% higher sensitivity than traditional gold-film sensors [37]. Rahman et al. coated a Au layer with graphene in a prism-waveguide SPR biosensor, increasing SPR angle variation by 40% and significantly enhancing the angular sensitivity [6]. Verma et al. inserted a Si layer between Au and graphene, achieving an angular sensitivity of 134.6 °/RIU by optimizing Au and Si thicknesses [38]. Therefore, 2D materials like graphene hold broad prospects in optical biosensing.

The prism-coupled Otto configuration serves as an effective structure for exciting SPR, where a thin air gap between the prism and the graphene-based sensing layer enables precise phase-matching conditions for enhanced light–matter interaction in the THz regime [39]. This paper presents a THz SPR biosensor based on a graphene-coupled prism Otto structure, where graphene conductivity is controlled via an external magnetic field. Replacing traditional metals with graphene to excite SPR, the sensor achieves higher sensitivity and tunability by adjusting magnetic field intensity. Additionally, graphene’s surface conductivity can be modulated by adjusting the position of the Fermi level to regulate sensitivity and quality factor. By adjusting the relevant parameters (e.g., external magnetic field intensity, dielectric layer thickness), the phase detection sensitivity of the biosensor can reach $3.1043\times 10^{5}$ deg RIU^−1^ for liquid sensing and $2.5854\times 10^{4}$ deg RIU^−1^ for gas sensing, demonstrating potential in chemical detection and medical diagnosis.

## 2. Theoretical Model and Method

**Figure 1:**
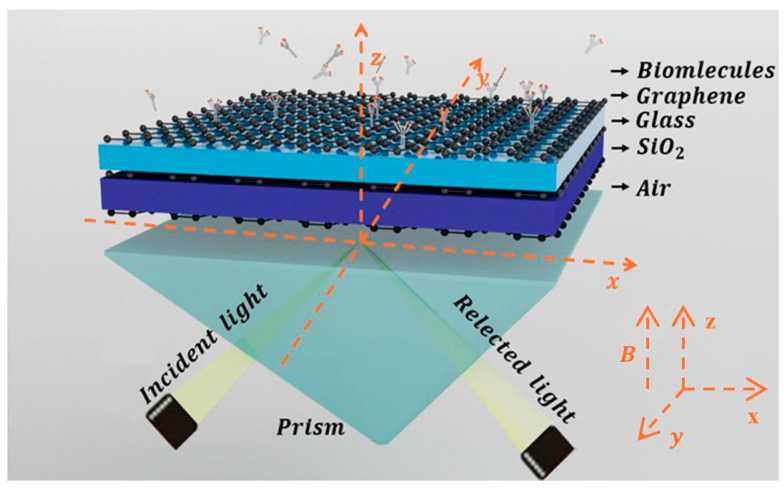
Schematic diagram of THz SPR biosensor based on graphene-coupled prism Otto structure.

The proposed Otto structure based on graphene-coupled prism consists, from bottom to top, of a coupling prism, graphene, SiO_2_, graphene, glass, graphene, and a sensing layer. Meanwhile, air is used as the intermediate medium between the coupling prism and the graphene layer, as shown in Figure 1. In this structure, silicon (Si) is selected as the material for the coupling prism. The dielectric constants of Si, glass, air, and SiO_2_ are set as $\varepsilon_{Si}$ = 12, $\varepsilon_{Glass}=2.25$, $\varepsilon_{Air}=1$, $\varepsilon_{SiO_{2}}=4$ [40], respectively. Assuming the incident light has a frequency of 3.2 THz, it enters graphene from one side of the coupling prism (passing through air first) and is then reflected from the other side. There is an air gap between the coupling prism and the first graphene layer to separate Si from graphene, with this distance set as $d_{Air}=5$ μm. The thickness of the SiO_2_ and glass layers are denoted as $d_{1}$ and $d_{2}$, respectively.

In optical sensing calculations, for the multilayer structure, we usually adopt the Transfer Matrix Method (TMM) [41] to calculate the transmittance (*T*) and reflectance (*R*) of the entire structure. Using TMM, we can compute the spectrum of the THz SPR biosensor based on the graphene-coupled prism Otto structure. In our simulations, the graphene layers were modeled as ideal single layers—a simplification adopted to establish a clear theoretical baseline and elucidate the fundamental plasmonic enhancement mechanisms, without introducing excessive complexity from structural variations. This approach aligns with common practice in theoretical studies of 2D material-enhanced sensors. While real graphene typically exhibits multi-layer domains and diverse defects (e.g., vacancies, grain boundaries, adatoms) that can alter optoelectronic properties and impact device performance—an observation also discussed in the recent experimental work by Mousavi-Kiasari et al.—explicitly incorporating these defects remains challenging [42]. This is due to the broad variety of defect types and the high computational cost of atomistically representing their effects in a multilayer system. For this reason, we focused on a tractable model featuring three monolayer graphene sheets, leveraging layer-wise conductivity summation as a reasonable approximation under conditions of minimal interlayer coupling. We fully acknowledge the implications of such non-idealities and plan to address them in future experimental studies using Chemical Vapor Deposition (CVD)-synthesized graphene, where defect distributions and layer uniformity can be characterized and directly correlated with sensor performance. When a uniform magnetic field acts vertically on graphene, its surface conductivity can be expressed as [34,43]:

(1) $$\bar{\sigma }=\left(\begin{array}{cc} \sigma_{xx} & \sigma_{xy}\\ \sigma_{yx} & \sigma_{yy} \end{array}\right),$$

There [42,44]:

$$\sigma_{xx}=\frac{e^{2}v_{f}^{2}|eB|\hbar (\omega +2i\Gamma )}{i\pi }\times \sum_{n=0}^{\infty }\{\frac{1}{M_{n+1}-M_{n}}\times \frac{n_{F}(M_{n})-n_{F}(M_{n+1})+n_{F}(-M_{n+1})-n_{F}(-M_{n})}{(M_{n+1}-M_{n}{)}^{2}-\hbar^{2}(\omega +2i\Gamma {)}^{2}}+(M_{n}\to -M_{n})\}$$

The input and output field coefficients are related via the total transfer matrix *M:*

(2) $$\left(\begin{array}{c} a_{1}\\ b_{1}\\ c_{1}\\ d_{1} \end{array}\right)=D_{1,2}P\left(d_{1,2}\right)D_{2,3}P\left(d_{2,3}\right)\ldots P\left(d_{N-1,N}\right)D_{N,N+1}\left(\begin{array}{c} a_{N+1}\\ b_{N+1}\\ c_{N+1}\\ d_{N+1} \end{array}\right)=M\left(\begin{array}{c} a_{N+1}\\ b_{N+1}\\ c_{N+1}\\ d_{N+1} \end{array}\right),$$

where matrix $D_{1,2}\;$is defined as

$$D_{1,2}=\frac{1}{2}\left(\begin{array}{cccc} \left(1+\frac{k_{2z\varepsilon_{1}}}{k_{1z\varepsilon_{2}}}+\frac{k_{2z}\sigma_{xx}}{\omega \varepsilon_{0}\varepsilon_{2}}\right) & \left(1-\frac{k_{2z\varepsilon_{1}}}{k_{1z\varepsilon_{2}}}-\frac{k_{2z}\sigma_{xx}}{\omega \varepsilon_{0}\varepsilon_{2}}\right) & \sigma_{xy} & \sigma_{xy}\\ \left(1-\frac{k_{2z\varepsilon_{1}}}{k_{1z\varepsilon_{2}}}+\frac{k_{2z}\sigma_{xx}}{\omega \varepsilon_{0}\varepsilon_{2}}\right) & \left(1+\frac{k_{2z\varepsilon_{1}}}{k_{1z\varepsilon_{2}}}-\frac{k_{2z}\sigma_{xx}}{\omega \varepsilon_{0}\varepsilon_{2}}\right) & \sigma_{xy} & \sigma_{xy}\\ \frac{\mu_{0}k_{2z}\sigma_{yx}}{k_{1z}\varepsilon_{0}\varepsilon_{2}} & -\frac{\mu_{0}k_{2z}\sigma_{yx}}{k_{1z}\varepsilon_{0}\varepsilon_{2}} & \frac{\mu_{0}\omega }{k_{1z}}(\frac{k_{1z}+k_{2z}}{\omega \mu_{0}}+\sigma_{yy}) & \frac{\mu_{0}\omega }{k_{1z}}(\frac{k_{1z}-k_{2z}}{\omega \mu_{0}}+\sigma_{yy})\\ -\frac{\mu_{0}k_{2z}\sigma_{yx}}{k_{1z}\varepsilon_{0}\varepsilon_{2}} & \frac{\mu_{0}k_{2z}\sigma_{yx}}{k_{1z}\varepsilon_{0}\varepsilon_{2}} & \frac{\mu_{0}\omega }{k_{1z}}(\frac{k_{1z}-k_{2z}}{\omega \mu_{0}}-\sigma_{yy}) & \frac{\mu_{0}\omega }{k_{1z}}(\frac{k_{1z}+k_{2z}}{\omega \mu_{0}}-\sigma_{yy}) \end{array}\right),$$

Here, $k_{1z}$ and *q* are the *z*- and *x*- components of the wave vector $k_{1}=\sqrt{\varepsilon_{1}}\omega /c$ in dielectric 1, with $k_{1z}=\sqrt{\varepsilon_{1}{\left(\omega c\right)}^{2}-q^{2}}$ and $k_{2z}=\sqrt{\varepsilon_{2}{\left(\omega c\right)}^{2}-q^{2}}$ (ω: angular frequency; *c*: speed of light in vacuum). Reflectance (*R*) and transmittance (*T*) are calculated as:

(3) $$\begin{array}{c} R_{TM}=|b_{1}{|}^{2},\\ R_{TE}=((\frac{k_{1z}}{\omega \mu_{0}}{)}^{2}+(\frac{q}{\omega \mu_{0}}{)}^{2})|d_{1}{|}^{2},\\ T_{TM}=|a_{N+1}{|}^{2},\\ T_{TE}=((\frac{k_{N+1z}}{\omega \mu_{0}}{)}^{2}+(\frac{q}{\omega \mu_{0}}{)}^{2})|c_{N+1}{|}^{2},\\ T=T_{TM}+T_{TE},\\ R=R_{TM}+R_{TE}, \end{array}$$

There,

$$\begin{array}{l} b_{1}=\frac{M_{31}M_{23}-M_{21}M_{33}}{M_{13}M_{31}-M_{11}M_{33}},\\ d_{1}=\frac{M_{43}M_{31}-M_{41}M_{33}}{M_{13}M_{31}-M_{11}M_{33}}, \end{array}$$

In this paper, phase detection sensitivity is defined as:

(4) $$S=\frac{\Delta \varphi }{\Delta n},$$

where Δφ is the differential phase between TM-polarized light and TE-polarized light. $\Delta \varphi =\left|\Delta \varphi_{TM}-\Delta \varphi_{TE}\right|$. SPR is excited by TM-polarized light in Otto structure, with TE-polarized light serving as a reference.

## 3. Results and Discussion

This section systematically analyzes the theoretical sensing performance of the proposed sensor. It should be noted that although experimental verification usually involves more practical factors (especially challenges in micro-nano scale fabrication), due to the limitations of current experimental conditions, this research phase mainly focuses on theoretical modeling and simulation analysis. It is worth pointing out that the sensing technology based on SPR has developed a variety of mature experimental protocols; therefore, the specific experimental implementation will be the focus of our subsequent research work. It should also be noted that in terms of material preparation, this study plans to use CVD to fabricate large-area, high-quality graphene films on metal substrates [45,46]. The specific process includes the following steps [47]: First, polycrystalline copper foil is converted into single-crystal Cu (111) foil through electroplating and high-temperature annealing, and further alloyed to form Cu–Ni (111) foil. Subsequently, the alloy substrate is heated to a specific temperature in the range of 1000 K–1320 K in the CVD system, and ethylene is introduced as the carbon source for graphene growth at the same time. After the completion of graphene growth, the electrochemical exfoliation method is intended to be used to transfer it to the Otto configuration substrate, so as to prepare for the subsequent experimental testing of SPR sensing performance.

**Figure 2:**
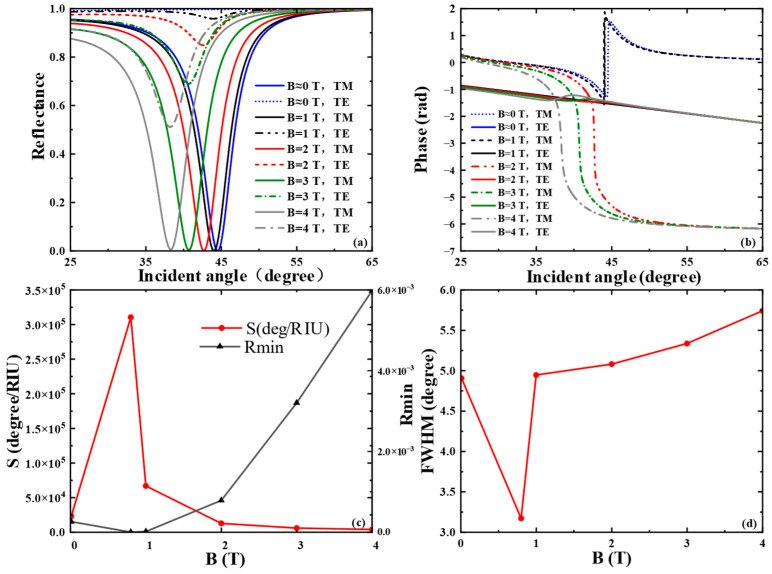
When the frequency of the excitation light source is 3.2 THz, the thicknesses of the SiO_2_ and the Glass layers are 5.1 μm and 5.0 μm, and the refractive index of the biosensing medium *n* = 1.3330: (**a**) Reflectance and (**b**) differential phase variation with magnetic field intensity *B*; (**c**) effects of different magnetic field intensity *B* on the phase detection sensitivity and minimum reflectance $R_{min}$ of the SPR curves; (**d**) effects of different magnetic field intensity *B* on the FWHM of the SPR curve.

In this paper, we first examined the effect of external magnetic field intensity (*B*) on the spectral reflectance and phase of the SPR curve, as depicted in Figure 2a,b. In our simulations, an external magnetic field was applied perpendicularly to the graphene plane (Figure 1), a specific orientation critical for activating pronounced magneto-optical effects. When aligned perpendicular to the surface, the magnetic field breaks time-reversal symmetry and induces significant off-diagonal components in the conductivity tensor, leading to measurable alterations in the SPR response, such as resonance shifts and polarization rotation. By contrast, a parallel magnetic field exerts negligible influence in this configuration, as it fails to elicit substantial magneto-optical activity. For the liquid biosensor, the refractive index of the sensing medium was set to 1.3330, which corresponds to typical biosensing conditions such as the detection of small molecules or protein binding events in aqueous solutions. The sharp resonance dip in Figure 2a is clearly attributed to the theoretical excitation of SPR at the graphene–dielectric interface, as demonstrated by our simulations. This is confirmed by the distinct, narrow reflectance minimum under TM polarization with a calculated value of $3.6543\times 10^{-7}$, indicating efficient energy transfer to graphene plasmon-polaritons. Notably, this feature is completely absent under TE polarization in our theoretical model—critical evidence that the observed phenomenon is a polarization-dependent plasmonic resonance rather than a general optical effect. The excitation is further validated quantitatively by the calculated Full Width at Half Maximum (FWHM) in Figure 2d, consistent with established theoretical models for graphene–dielectric SPR modes. When *d*_1_ and *d*_2_ are 5.1 μm and 5.0 μm, respectively, as *B* grows from 0 T to 4 T, the resonance angle of the SPR curve shifts slowly to the left due to the wave vector matching between the incident light and the surface plasmon wave. The phase curve of SPR undergoes a sharp phase jump at the resonance angle corresponding to the minimum reflectance. When *B* = 1 T, the THz SPR biosensor exhibits the maximum phase jump. By integrating the sensor structure and optimizing the thicknesses of the SiO_2_ layer and the Glass layer, stronger SPR can be excited as much as possible. In addition, to better characterize the performance of the proposed SPR optical biosensor, we investigated the phase detection sensitivity and the FWHM of the SPR curve. The minimum reflectance *R*_min_ of the SPR curve is shown in Figure 2c. At a magnetic field intensity of *B* = 1 T, a relatively small reflectance $R_{min}$($1.4342\times 10^{-5}$) can be obtained, where the SPR excitation is stronger and the phase detection sensitivity reaches 6.6720 × 10^4^ $degRIU^{-1}$. By optimizing the parameters, the reflectance is minimized to $3.6543\times 10^{-7}$ at a magnetic field intensity B=0.8 T and △n=0.0005, and the phase detection sensitivity reaches a maximum of $3.1043\times 10^{5}$ $deg\;RIU^{-1}$. As shown in Figure 2d, the FWHM curve is minimized to 3.17° at *B* = 0.8 T; thereafter, the FWHM gradually rises with the increase in magnetic field intensity, indicating that the sensor has good accuracy.

**Figure 3:**
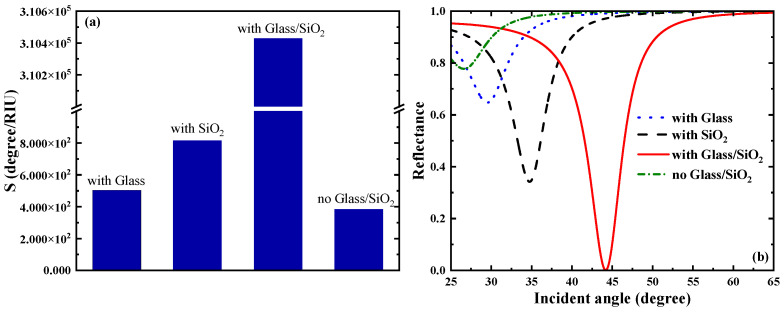
(**a**) Comparison plot of the effect of the presence or absence of a dielectric layer on the sensitivity of the sensor. (**b**) Effect of the presence or absence of a dielectric layer on the SPR reflectance curve.

Going forward, we analyzed the impact of the dielectric layers (Glass and SiO_2_) on the sensor. From Figure 3a, the sensitivity is 502.75 $degRIU^{-1}$ when the dielectric layer is only the Glass layer; the sensitivity is 815.28 $degRIU^{-1}$ when the dielectric layer is only the SiO_2_ layer; when there are two dielectric layers (Glass and SiO_2_), the sensitivity reaches $3.1043\times 10^{5}\;degRIU^{-1}$; and when there is no dielectric layer, the sensitivity is 383.31 $degRIU^{-1}$. It can be seen from Figure 3b that the resonance between the evanescent wave and the surface plasmon wave of the medium is weak when there is only a single layer of medium, whereas a sharp reflection peak gradually appears in the SPR reflectance curve when there are two layers of medium, at which point the SPR excitation is the strongest.

**Figure 4:**
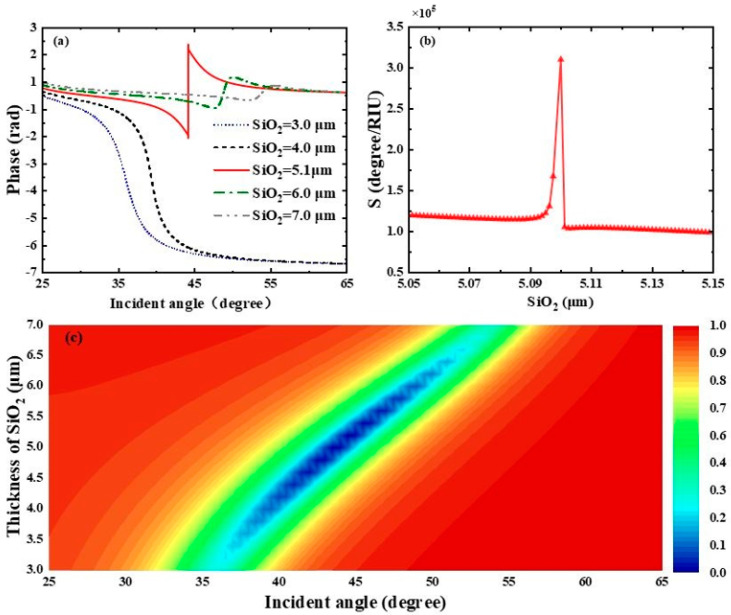
(**a**) Differential phase of the biosensor with respect to the incident angle when the refractive index of the biosensing medium is 1.333. (**b**) Variation curve of the phase detection sensitivity of the proposed biosensor under different SiO_2_ thicknesses. (**c**) Plotted reflectance spectra of the biosensor as a function of incident angle and SiO_2_ layer thickness.

To further figure out the effect of dielectric layers on the performance of this SPR biosensor, we first focused on the influence of the SiO_2_ layer thickness on the reflectance spectra. As clearly shown in Figure 4a, the differential phase of the biosensor shows a sharp phase jump when the thickness of the SiO_2_ layer is 5.1 μm. At this thickness, the biosensor displays the minimum reflectance $3.6543\times 10^{-7}$ and the highest phase detection sensitivity, reaching $3.1043\times 10^{5}$$\;degRIU^{-1}$. By observing Figure 4b, the interaction between biomolecules and the sensor gradually weakens as the thickness of the SiO_2_ layer increases. When the thickness of the SiO_2_ layer exceeds 6.5 μm, the reflectance grows with the increase in SiO_2_ thickness, and even total reflection occurs. The phase detection sensitivity reaches a maximum at a SiO_2_ thickness of 5.1 μm and then decreases rapidly. We also calculated the reflectance spectra related to the SiO_2_ layer thickness of the THz SPR biosensor based on the graphene-coupled prism Otto structure using TMM, as shown in Figure 4c.

**Figure 5:**
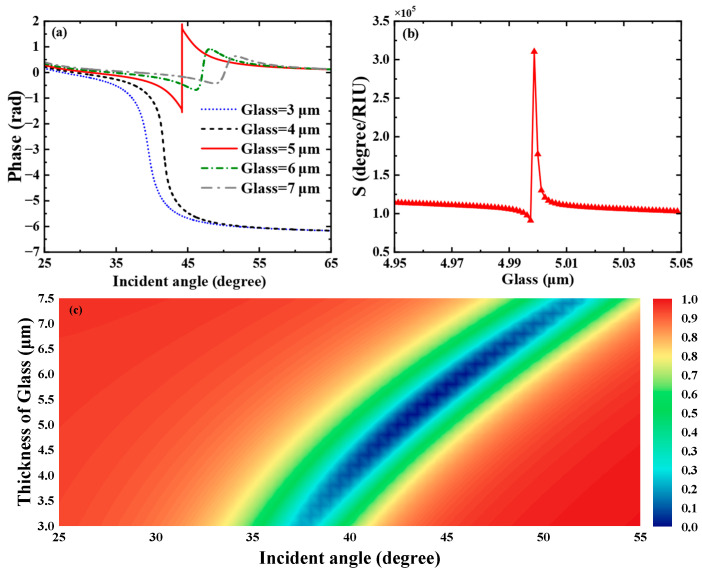
(**a**) Differential phase with respect to the incident angle when the refractive index of the biosensing medium is set to 1.333. (**b**) Variation in the phase detection sensitivity of the proposed biosensor with respect to the thickness of the Glass layer. (**c**) Reflectance spectra of the biosensor as a function of incident angle and Glass layer thickness.

**Figure 6:**
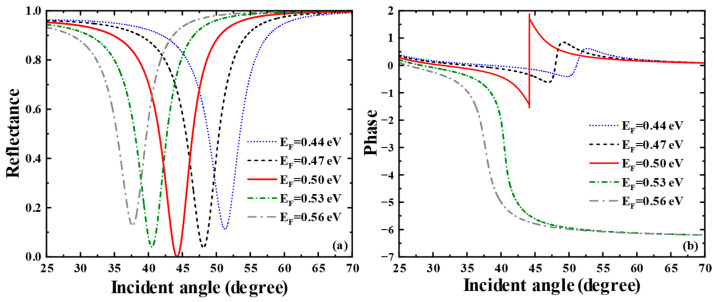
(**a**) Curves of reflectance versus incident angle when the Fermi energy levels of graphene are taken as $E_{F}$ = 0.44 eV, $E_{F}$ = 0.47 eV, $E_{F}$ = 0.50 eV, $E_{F}$ = 0.53 eV, $E_{F}$ = 0.56 eV, respectively. (**b**) Curves of phase versus incident angle when the Fermi energy levels are taken as $E_{F}$ = 0.44 eV, $E_{F}$ = 0.47 eV, $E_{F}$ = 0.50 eV, $E_{F}$ = 0.53 eV, $E_{F}$ = 0.56 eV, respectively.

Subsequently, we profoundly unearthed the effect of the Glass layer thickness on the THz SPR biosensor based on the graphene-coupled prism Otto structure. As shown in Figure 5a, which displays the sensitivity of the Heaviside stepwise jump of the differential phase, a sharp phase jump occurs when the Glass layer thickness is 5.1 μm. At this thickness, the biosensor achieves a minimum reflectance of $3.6543\times 10^{-7}$ and the phase detection sensitivity reaches the maximum of $3.1043\times 10^{5}$$\;degRIU^{-1}$. The variation curve of phase detection sensitivity with Glass layer thickness is shown in Figure 5b, where the phase detection sensitivity reaches its maximum at a Glass layer thickness of 5.0 μm and then decreases rapidly. As shown in Figure 5c, we also calculated the reflectance spectra related to the thickness of the Glass layer of this SPR biosensor using TMM. The parametric properties of materials can inform our search for sensing solutions with higher sensitivity and provide references for achieving dynamically tunable sensitivity characteristics. Hence, we deeply discuss the impact of graphene’s parametric properties on the overall performance of the sensor. First, we pay attention to the effect of variations in graphene’s Fermi energy level on the sensing structure. As shown in Figure 6a, we present the curves of the sensor’s reflectance versus incident angle when the graphene Fermi energy levels are $E_{F}$ = 0.44 eV, $E_{F}$ = 0.47 eV, $E_{F}$ = 0.50 eV, $E_{F}$ = 0.53 eV, $E_{F}$ = 0.56 eV, respectively. Figure 6b reflects the phase variations in the sensor at these Fermi energy levels. Meanwhile, the corresponding phase detection sensitivities at each Fermi energy level are calculated as 349.56 $degRIU^{-1}$, 1178.9 $degRIU^{-1}$, $3.1043\times 10^{5}$ $degRIU^{-1}$, 2062.5 $degRIU^{-1}$, 1861.1 $degRIU^{-1}$. From Figure 6a, it can be seen that as the Fermi energy level gradually climbs within the range of 0.47 eV to 0.56 eV, the reflection peaks first narrow, then broaden, and the corresponding excitation intensity of SPR changes from weak to strong and then to weak. In Figure 6b, the phase of the SPR curve reveals a sharp jump at the resonance angle corresponding to the minimum reflectance. At $E_{F}$ = 0.5 eV, the SPR biosensor demonstrates the largest phase jump. At this point, the reflectance is the sharpest, the excitation capability of SPR is the strongest, and the sensitivity reaches a maximum of $3.1043\times 10^{5}$$\;degRIU^{-1}$.

In addition, we have comprehensively compared the structure of the proposed SPR biosensor with those of 2D material-enhanced SPR biosensors reported in other literature, as shown in Table 1. A key consideration for the practical adoption of any sensor technology lies in its fabrication complexity. While the devices reported in Refs. [48,49,50,51] exhibit high sensitivity, they rely on precise nanoscale layering (e.g., 4 nm Si in [48], few-layer BP and graphene in [49], and atomically thin α-MoO_3_ in [51]), rendering their consistent reproduction challenging and costly outside specialized laboratories. In contrast, our proposed device features a structure highly compatible with well-established microfabrication techniques such as photolithography. Its key layers can be patterned with precision, and the entire device could be reliably mass-produced, potentially through collaborations with commercial foundries. Compared to atomically precise methods, this approach significantly eases fabrication tolerances, enabling the sensor to transition from a laboratory prototype to a manufacturable product. Notably, our design not only simplifies the manufacturing process but also maintains competitive sensitivity, outperforming several nanoscale-based sensors (e.g., [49,50]). This unique combination of performance and fabricability makes our sensor particularly well-suited for real-world applications where robustness, cost-effectiveness, and scalability are critical. Through various comparisons, it is evident that our proposed scheme achieves higher sensitivity than previous SPR biosensors while featuring a relatively simple structure.

**Figure 7:**
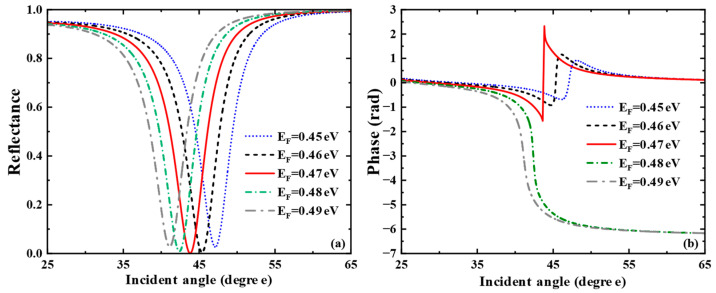
For Air sensing: (**a**) Curve of reflectance versus incident angle when the Fermi energy level of graphene varies from 0.45 eV to 0.49 eV. (**b**) Curves of phase versus incident angle when the Fermi energy levels are taken as $E_{F}$ = 0.45 eV, $E_{F}$ = 0.46 eV, $E_{F}$ = 0.47 eV, $E_{F}$ = 0.48 eV, $E_{F}$ = 0.49 eV, respectively.

The above discussion keeps a watchful eye on the application in liquid sensing. By adjusting parameters, we can further explore the sensing performance when the sensing medium is gas. We consider the sensitivity characteristics of the gas sensor when the refractive index of the sensing medium changes from 1.000 to 1.0005, simulating the detection of ambient atmospheric gases such as carbon dioxide or methane. Here, we only present the research on the influence of graphene’s Fermi energy level. As shown in Figure 7a, when the Fermi energy level changes from 0.44 eV to 0.48 eV, the reflection peak first narrows, then broadens, and the corresponding SPR excitation ability changes from weak to strong and then to weak. In Figure 7b, the phase of the SPR curve has a sharp jump at the resonance angle corresponding to the minimum reflectance. In the meantime, we calculated the corresponding phase detection sensitivities at each Fermi energy level as 1992.8 $degRIU^{-1}$, 4779.7 $degRIU^{-1}$, $2.5854\times 10^{4}$$\;degRIU^{-1}$, 4175.2 $degRIU^{-1}$ and 2445.9 $degRIU^{-1}$. The SPR biosensor exhibits the largest phase jump at $E_{F}=0.47$ eV. At this point, the SPR excitation is the strongest, and the sensitivity of the gas biosensor reaches a maximum of $2.5854\times 10^{4}$$\;degRIU^{-1}.$

## 4. Conclusions

In this paper, we propose a THz SPR biosensing scheme based on a graphene-coupled prism Otto structure. Using TMM, the reflectance and transmittance of graphene are calculated. This sensor can achieve magnetic control by regulating the conductivity of graphene through an external magnetic field. In addition, the coupling prism, together with graphene, SiO_2_, and Glass layers, couples to excite SPR, generating sharp reflection peaks, which can be dynamically adjusted by changing the relevant parameters of graphene. By optimizing structural parameters such as the thickness of the SiO_2_ layer, the thickness of the Glass layer, and the conductivity of graphene, we find that when the sensor is used for liquid sensing, its minimum reflectance is 3.6543 × 10^−7^, and the phase detection sensitivity is as high as 3.1043 × 10^5^ deg RIU^−1^; when used for gas sensing, the phase detection sensitivity reaches 2.5854 × 10^4^ deg RIU^−1^. Compared with traditional SPR biosensors, this sensing scheme has a simpler structure, lower requirements for manufacturing processes, and can achieve tunability and higher sensitivity. We hold the belief that this scheme has potential application value in chemical detection, medical diagnosis and other fields.

## Figures and Tables

**Table 1:** Comparison of recent literature on SPR sensing performance enhancement methods based on different nanostructures.

References	Structure	Sensitivity (deg RIU^−1^)	Frequency	Mechanism
[48]	MoSe_2_/silicon/Au	$1.1\times 10^{7}$	THz	SPR
[49]	BP/Graphene	$7.49\times 10^{4}$	THz	SPR
[50]	SF11/Au/BlueP/Graphene	$1.43\times 10^{5}$	THz	SPR
[51]	SF11/Au/α-MoO_3_/Graphene	$1.5172\times 10^{5}$	THz	SPR
This work	SiO_2_/Graphene/Glass	$3.1043\times 10^{5}$	THz	SPR

## Data Availability

The original contributions presented in this study are included in the article. Further inquiries can be directed to the corresponding authors.
